# Perovskite-Compatible Electron-Beam-Lithography Process Based on Nonpolar Solvents for Single-Nanowire Devices

**DOI:** 10.1021/acsanm.2c00188

**Published:** 2022-02-22

**Authors:** Nils Lamers, Zhaojun Zhang, Jesper Wallentin

**Affiliations:** Synchrotron Radiation Research and NanoLund, Department of Physics, Lund University, Box 124, Lund 22100, Sweden

**Keywords:** CsPbBr_3_, perovskite, electron-beam lithography, nanowire, patterning

## Abstract

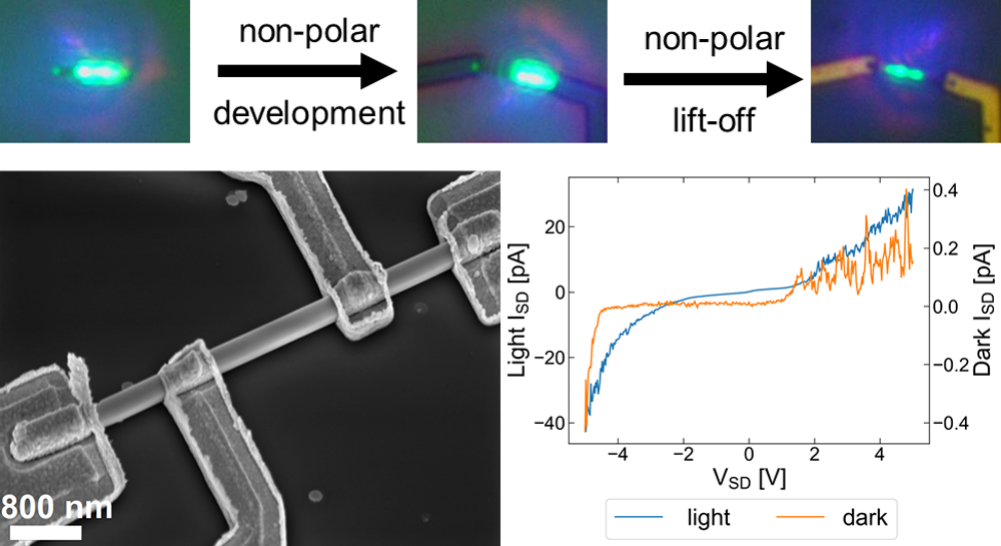

Metal halide perovskites (MHPs) have been studied intensely as the active material for optoelectronic devices. Lithography methods for perovskites remain limited because of the solubility of perovskites in polar solvents. Here, we demonstrate an electron-beam-lithography process with a poly(methyl methacrylate) resist based on the nonpolar solvents *o*-xylene, hexane, and toluene. Features down to 50 nm size are created, and photoluminescence of CsPbBr_3_ nanowires exhibits no degradation. We fabricate metal contacts to single CsPbBr_3_ nanowires, which show a strong photoresponsivity of 0.29 A W^–1^. The presented method is an excellent tool for nanoscale MHP science and technology, allowing for the fabrication of complex nanostructures.

## Introduction

Metal halide perovskites (MHPs) have attracted increased research attention because of their optoelectronic properties, most notably spurred on by the rapid efficiency improvements in solar cells.^1^ Optoelectronic devices such as light-emitting diodes,^2,3^ X-ray scintillators,^4,5^ photodetectors,^6−10^ and others based on MHPs have also shown promise for low-cost and flexible next-generation devices. A major advantage of MHPs is the possibility of solution-based processing, which allows low-cost crystal growth, especially compared to materials like III–V semiconductors. However, the high solubility of MHPs in polar solvents^11−13^ also comes with major limitations to the techniques that can be used in the manufacturing of nanoscale devices because standard nanofabrication techniques make frequent use of polar solvents, like water and acetone.

A lack of lithographic techniques that exclusively use nonpolar solvents is one of the biggest hindrances to the fabrication of nanoscale MHP devices. Without such techniques, the nanostructuring of both MHP-active and contact layers is not possible. Especially for contacts, low-resolution shadow masking techniques are often used.^14^ Recently, advancements have been made toward adapting established lithography techniques for use with perovskites. Nanostructured perovskite has been produced via nanoimprint lithography,^15,16^ ultraviolet-light lithography,^11,13,17^ and electron-beam lithography (EBL).^18,19^ EBL has been carried out on MHPs commonly using a poly(methyl methacrylate) (PMMA) resist.^12,19−21^ PMMA itself is suitable because it is readily commercially available in nonpolar solvents like chlorobenzene and anisole and highly soluble in many other nonpolar solvents.^22^ Zhang et al.^19^ and Yang et al.^20,21^ used 1:3 methyl isobutyl ketone/isopropyl alcohol (MIBK/IPA) developer solutions, a developer commonly used for PMMA, despite its polarity and potential to dissolve MHPs. To mitigate the damage to the MHP, Zhang et al. used only a 5 s development time, while Yang et al. dried the developer solution thoroughly enough to not dissolve CsPbBr_3_, but neither approach was used to fabricate contacts via a lift-off process. In contrast, Lin et al.^12^ used a mixture of chlorobenzene and hexane (1:3) to develop and evaporate 90-nm-thick metal contacts. However, we found this method to be inadequate for contacting CsPbBr_3_ nanowires (NWs) with a metal of sufficient thickness.

Here, we present and characterize an MHP-compatible PMMA process based on the nonpolar solvents *o*-xylene, hexane, and toluene.^23,24^ The *o*-xylene/hexane-based developer shows a development performance similar to that of the widely accepted 1:3 MIBK/IPA solution and displays the ability to produce line arrays with 250 nm pitch and 50 nm single lines. Additionally, our process does not use chlorinated solvents, making it more environmentally friendly and reducing the risk of unintentional anion-exchange processes that can occur with chlorinated solvents.^25,26^ Using this process, CsPbBr_3_ single-NW (diameter, 150–350 nm; length, 1–10 μm) devices were successfully fabricated in two- and four-probe geometries. The excellent photoresponse of our devices demonstrates the feasibility of our compatible PMMA process for the fabrication and development of MHP nanoelectronic devices.

## Results and Discussion

**Figure 1:**
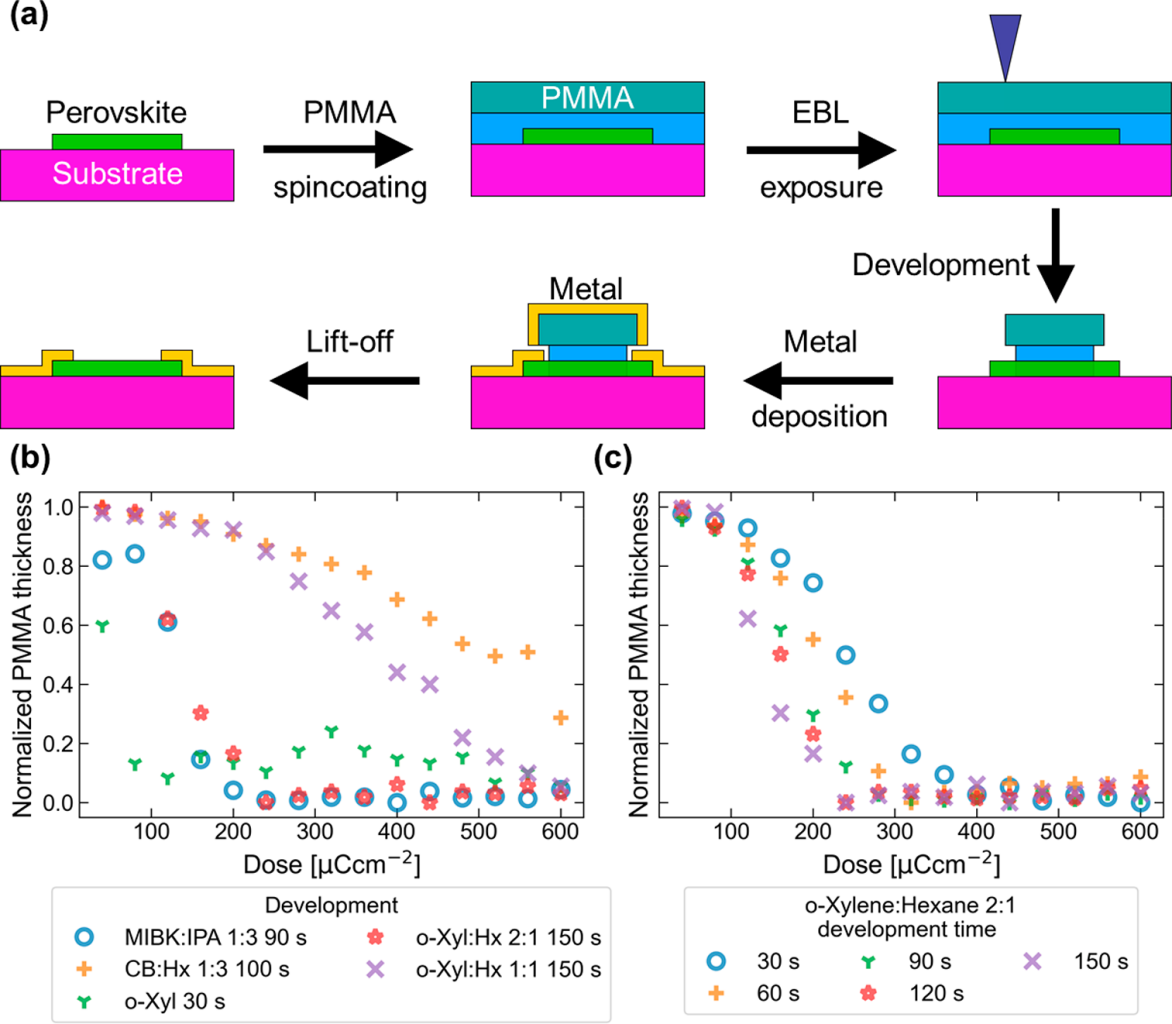
(a) PMMA-bilayer EBL processing scheme. First, a PMMA bilayer is deposited on a perovskite sample via spin-coating. The EBL pattern is then written using an EBL tool and transferred to the PMMA layer by immersion in a developer solution. An undercut is created in this step because of the higher solubility of the bottom PMMA layer and the blooming of the focused electron beam inside the resist. Metal is then deposited and lifted off by immersion in a remover, which dissolves all PMMA. (b) Comparison of the normalized PMMA height for different developer solutions for doses of 40–600 μC cm^–2^. The normalized height is calculated as the ratio between the heights of exposed and as-deposited PMMA (0 is full development, and 1 is no development). (c) Normalized PMMA height for samples developed with a 2:1 *o*-xylene/hexane developer for different development times.

A general scheme for PMMA EBL processing is illustrated in Figure 1a. The overall process is standard, and the novelty lies in the developer solution. A PMMA bilayer is deposited via spin-coating, and then the desired pattern is written using an EBL tool. Electron-beam exposure locally increases the solubility of the PMMA film. Development transfers the written pattern to the PMMA resist layer by selectively dissolving exposed PMMA and, for the bilayer, creates an undercut. For metal patterning, a metal film is deposited and the remaining PMMA is dissolved, lifting off any metal not deposited onto the revealed sample surface. For thick metal layers, the undercut profile of the bilayer increases the success of this final step. The PMMA process could also be used for patterning of the perovskite itself, for instance, using etching or ion milling, but this is not further explored here.

First, we tested the development behavior. A PMMA495C4 and PMMA950A5 dual-layer resist was spun onto silicon substrates and exposed to doses ranging from 40 to 600 μC cm^–2^. Development was then carried out in mixtures of 1:3 MIBK/IPA and 1:3 chlorobenzene/hexane and several *o*-xylene/hexane mixtures (1:0, 2:1, 1:1, and 1:2) and the remaining resist height measured with a profilometer. All chemicals were used as-received without further purification. The normalized PMMA height after development was calculated from the known height of the as-deposited resist film and the measured feature depths. This indicates the development quality because a good developer will only dissolve all exposed PMMA, whereas a poor developer will dissolve unexposed areas or fail to fully dissolve the exposed resist. The normalized PMMA height for selected times is shown in Figure 1b. The pure *o*-xylene developer is the most sensitive, allowing for full development even at low doses of 80 μC cm^–2^ and short development times of 30 s. However, it is also the least selective because the remaining resist thickness on the sample measures only 360 nm compared to the nominal resist height of 420 nm. For development times longer than 30 s, *o*-xylene continues to dissolve unexposed PMMA, reducing the overall resist height and pattern fidelity. Both the standard 1:3 MIBK/IPA and the 2:1 *o*-xylene/hexane solution show very similar development behaviors with a clearing dose of around 240 μC cm^–2^ for development times of 90 and 150 s, respectively, and no appreciable loss in the overall resist height. While the clearing dose is quite similar, the surface roughness caused by PMMA residuals can be seen in the profilometer profile for 2:1 *o*-xylene/hexane up to a dose of around 400 μC cm^–2^. In contrast, the more diluted 1:1 *o*-xylene/hexane and 1:3 chlorobenzene/hexane solutions do not achieve full development within the tested dose and time ranges. Figure 1c shows the normalized PMMA height of the 2:1 *o*-xylene/hexane developer for different development times. Full development can be achieved for development times as low as 30 s at a dose of around 440 μC cm^–2^. While the exact combination of clearing dose and development time will also depend on the sample specific parameters (feature size and pitch, substrate material, and resist thickness), the wide process window shown by this solution should make it suitable for many applications.

**Figure 2:**
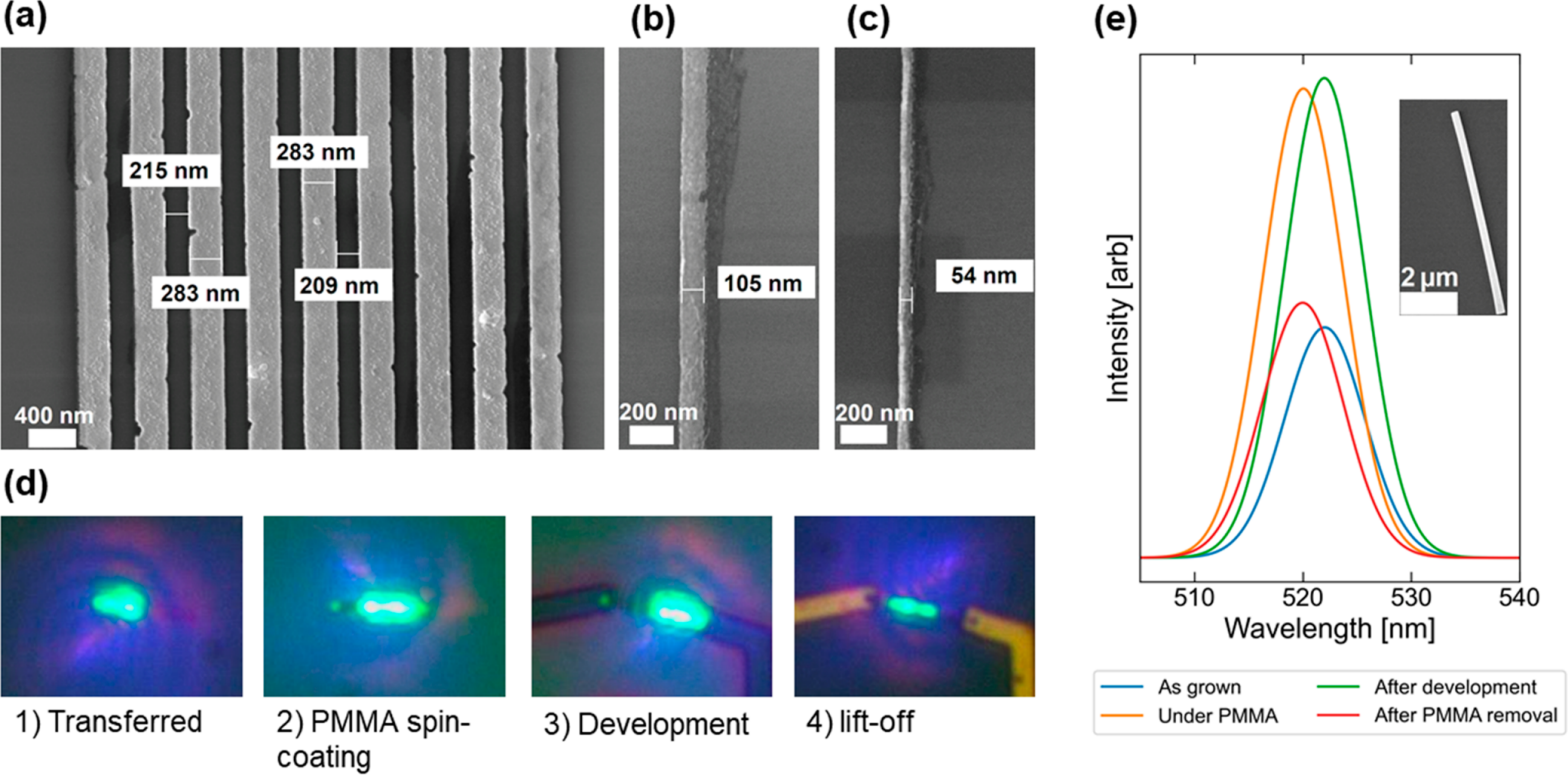
(a) 250 nm line array, (b) 100 nm single line, and (c) 50 nm single line created with the 2:1 *o*-xylene/hexane process. (d) Optical and PL image of a CsPbBr_3_ NW throughout device fabrication. A focused 5 mW, 395 nm laser was used for excitation. (e) Photoluminescence spectra of a NW throughout device fabrication. Here, an unfocused 485 nm laser spot with a power density of 2.29 mW cm^–2^ was used for excitation. The inset shows the SEM image of a transferred CsPbBr_3_ NW.

To test the resolution possible with this developer, line arrays and single lines were deposited. The 2:1 *o*-xylene/hexane process with a 120 s development time was used, followed by a 3 s dip in pure *o*-xylene to ensure clean development and enhance the undercut. A 30 nm layer of gold was deposited and lifted off by immersion in toluene at 60 °C. Line arrays with a line width of 250 nm are shown in Figure 2a and were written with a dose of 280 μC cm^–2^. Individual lines with widths as small as 50 and 100 nm (Figure 2b,c) could be created with doses of 360 and 400 μC cm^–2^, respectively. Line arrays with lower pitch all failed to lift-off correctly because of feature collapse, likely caused by the undercut and *o*-xylene dip necessary to obtain a clean substrate surface. While it may be possible to create these structures by using O_2_ plasma instead of the *o*-xylene dip to clean the developed surface, this is likely to cause damage to any underlying perovskite. Instead, the resist and metal thicknesses should be optimized for very small feature sizes, but this is beyond the scope of this initial report.

We investigated how the process affected the optical quality of CsPbBr_3_ NWs by recording photoluminescence (PL) spectra at each step of the process because PL is sensitive to defects. The optical images acquired with a focused 5 mW, 395 nm laser are shown in Figure 2d, while the spectra acquired with an unfocused 485 nm laser spot with a power density of 2.29 mW cm^–2^ are shown in Figure 2e. Only small shifts of about 5 nm in the PL peak position are observed. The PL intensity shows a slight nonsystematic variation, where the final intensity is marginally higher than the original one. However, this is most probably due to variations in the alignment of the NW to the laser excitation source, because the NW had to be realigned for each measurement, and not caused by actual changes in the optical quality. This result also indicates that no damage is done to the material by the electron beam at this acceleration voltage and exposure dose. Thus, we conclude that the process does not cause appreciable degradation of the optical quality of the NWs.

**Figure 3:**
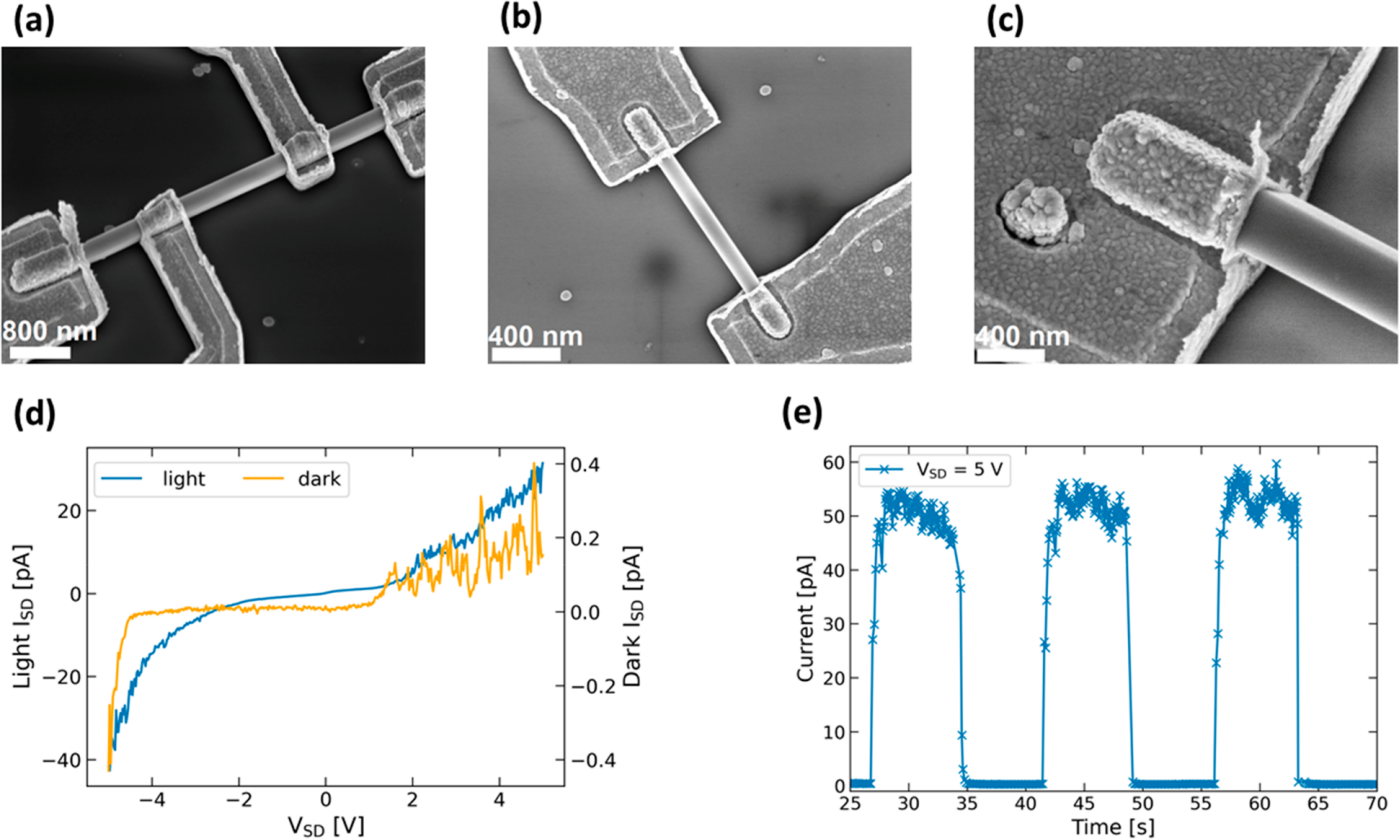
(a–c) SEM images of different single-NW devices manufactured using *o*-xylene-based EBL. Parts a and c were imaged at a 30° tilt. (d) Dark and light *I*–*V* curves of a device. (e) On/off photoresponse of the same device as that in part c at *V*_SD_ = 5 V.

Finally, we used the EBL patterning process to create single-NW devices. The 2:1 *o*-xylene/hexane process with a 120 s development time and a 3 s *o*-xylene dip was used again. For the contacts, 20 nm Ti and 200 nm Au were evaporated at an angle and under constant rotation to ensure sidewall coverage at the NW contact. Lift-off was carried out by immersion in toluene at 60 °C. Scanning electron microscopy (SEM) images of fabricated devices are shown in Figure 3a–c, and a typical set of dark and light *I*–*V* curves is shown in Figure 3d. This device displays nonlinear behavior with a very low dark current of 0.4 pA at *V*_SD_ = −5 V. Upon illumination with an optical power of *P* = 16 mW cm^–2^ of 395 nm UV light, the current increases to 40 pA at −5 V, which corresponds to a responsivity  = 0.29 A W^–1^ with an effective NW area of *A*_NW_ = 8.38 × 10^–9^ cm^–2^. The on/off photoresponse of the same device at *V*_SD_ = 5 V is shown in Figure 3e. When the device is turned on, the current stabilizes within 0.4 ms, with the off response being quicker than the 0.13 s time resolution of the electronics. Multiple cycles of photocurrent measurements for four different devices can be found in Figure S1. The devices show consistent behavior over the measurement period, with some variation settling within the first two measurement cycles. Furthermore, the variation between devices is quite small.

The nonlinear behavior could be by caused Schottky-like contacts^27^ or ion migration effects that screen the external electric field.^28−30^ The strong photoresponse observed for our devices indicates that the nonlinear and hysteresis-like *I*–*V* behavior is more likely to be caused by ion migration effects than poor nonohmic contacts. This is further supported by the photocurrent saturating at around 10 nA for *V*_SD_ = ±5 V for all measured devices, indicating a similar resistivity. If the contacts were nonohmic due to poor contact quality, the device-to-device variation would be expected to be much more significant. A full exploration of the complex electron- and ion-transport dynamics of these devices is beyond the scope of this paper, but we can conclude that our method can be used for the creation of nanoscale MHP electrical devices.

In conclusion, we have presented an EBL process based on nonpolar solvents, which are compatible with MHPs. The process has a large process window and can be used to create nanoscale structures. We use metal evaporation and lift-off to create NW devices, but the process should also be compatible with patterning of the MHP itself. Thus, our results allow for complex nanoscale MHP devices based on top-down processing.

## Supporting Information Available

Photocurrent *I*–*V* sweeps over multiple cycles for additional devices, SEM images of additional devices, and additional experimental details, materials, and methods (PDF)

## Abbreviations

EBL: electron-beam lithography
IPA: isopropyl alcohol
MHP: metal halide perovskite
MIBK: methyl isobutyl ketone
NW: nanowire
PMMA: poly(methyl methacrylate)
SEM: scanning electron microscopy
