# Supplementary material for: Perovskite-Compatible Electron-Beam-Lithography Process Based on Nonpolar Solvents for Single-Nanowire Devices
Source: ACS Appl Nano Mater. 2022 Feb 22;5(3):3177–82. doi: 10.1021/acsanm.2c00188 (PMC8961732; doi:10.1021/acsanm.2c00188)
Supplement: Supplementary file 1 — an2c00188_si_001.pdf [file an2c00188_si_001.pdf]

Supporting information:

Perovskite-Compatible Electron-Beam-Lithography Process Based on Nonpolar Solvents for Single-Nanowire Devices

Nils Lamers, Zhaojun Zhang, Jesper Wallentin*

Synchrotron Radiation Research and NanoLund, Department of Physics, Lund University, Box 124, Lund, 22100, Sweden.

*Email: nils.lamers@sljus.lu.se

Experimental

Chemicals. Ortho-xylene (o-xylene), toluene, chlorobenzene, and methyl isobutyl ketone (MIBK) were all purchased from Sigma-Aldrich. Hexane was purchased from VWR chemicals. Isopropanol (IPA) and acetone were purchased from MicroChemicals. PMMA495 C4 (in chlorobenzene) and PMMA950A5 (in anisole) resists were purchased from Kayaku Advanced Materials. 495 and 950 denote the chain length of the PMMA molecules, with shorter chains being more soluble. All chemicals were used as received without further purification.

Materials. Silicon substrates with 100 nm wet-grown SiO2 were purchased from Siegert Wafer and used for all samples. All substrates were diced to 3 mm by 5 mm pieces and cleaned by sonication in isopropanol and acetone before resist deposition or nanowire transfer in the case of device samples.

Resist processing. All experiments were carried out using a bilayer resist consisting of a PMMA495 bottom layer and PMMA950 top layer. Both layers were spun at 3000 rpm for 50 s with a 5 s ramp at 500 rpm, before baking on a hotplate at 60 °C for 20 min. The temperature of 60 °C is chosen as it is just below the phase transition temperature of $CsPbBr_3$ NWs [1].

Development test samples. EBL was carried out using a Raith 150 EBL system with 20 kV acceleration voltage using a 30 µm aperture (0.34 nA beam current). Exposure doses of 40 µC/cm^2 to 600 µC/cm^2 (40 µC/cm^{2}steps) were used to write 50 µm by 50 µm squares. All developers were mixed by volume shortly before use. This is especially important for the mixtures containing hexane, as the high volatility of hexane can lead to a fast change in volume ratio and therefore developer performance. Hexane was used as the rinse solvent for o-xylene, all o-xylene:hexane ratios (1:0, 2:1, 1:1, 1:2), and chlorobenzene:hexane 1:3. Isopropanol was used as the rinse solvent for MIBK:IPA developers. Samples were dried by blow-drying with N_2. Development times of 30 s, 60 s, 90 s, 120 s, and 150 s were tested for all developers.

Resolution test samples. EBL was carried out using a Raith 150 EBL system with 20 kV acceleration voltage using a 30 µm aperture (0.34 nA beam current). Line arrays and single lines (widths 1 µm, 500 nm, 250 nm, 100 nm, and 50 nm; line pitch = line width) were exposed at doses ranging from 200 µC/cm^2 to 500 µC/cm^2 using 20 µC/cm^2 steps. Samples were developed using o-xylene:hexane 2:1 for 120 s. To ensure full clean development and enhance the undercut, the samples were dipped into o-xylene for 3 s, swirled in the developer solution once and then rinsed in hexane again. The swirling in the developer solution is necessary to prevent PMMA dissolved in the o-xylene from falling out of solution on top of the sample upon rinsing in hexane, as this will dirty the pattern. 3 nm titanium and 30 nm gold were then evaporated using a Temescal e-beam evaporator. Lift-off was performed by immersing in 60 °C warm toluene, followed by rinsing in hexane.

$CsPbBr_3$ nanowire devices. $CsPbBr_3$ NWs (diameter: 150 nm – 350 nm, length: 1- 10 µm) were grown from a precursor of CsBr and PbBr2 in DMSO using an anodised aluminium membrane (AAO) template. The NWs were transferred onto substrates with per-patterned electrodes and a pre-patterned marker pattern using a clean room tissue, similar to III-V NW device processing [2]. EBL was carried out using a Raith 150 EBL system with 20 kV acceleration voltage using a 10 µm aperture (0.01 nA beam current) and 400 µC/cm^2 dose. Development was carried out identical to resolution test samples (s. above). Contacts (20 nm Ti / 200 nm Au) were deposited in the same evaporator at a 30° angle and with continuous

rotation. The angle and rotation are critical to ensure a continuous metal film around the contact area. Lift-off was performed in toluene at 60 °C, followed by rinsing in hexane.

Characterisation. Resist profiles were measured using a Bruker DekTak XT profilometer with a 2 µm tip at a scan speed of 1 µm/s (3.3 nm per data point). SEM images were taken using a Zeiss LEO SEM. Electrical measurements were performed with a Keithley 4200A-SCS parameter analyser and a Cascade 11000B probe station. A UV flashlight (395 nm, 16 mW/cm^2) was placed 6.5 cm from the device to measure the photoresponse.

PL measurements. Optical images of luminescing nanowires were obtained using a focused 375 nm CW laser with 5 mW power connected to a microscope and CCD camera. To obtain photoluminescence spectra, NWs were excited using diffuse 485 nm CW diode laser with power density 2.29 mW/cm^2 and the light passed through a grating in front of a Princeton Instruments CCD. The spectra were calculated from the distance between 0th order and 1st order diffraction spots. The system was calibrated using a 515 nm Ar laser.

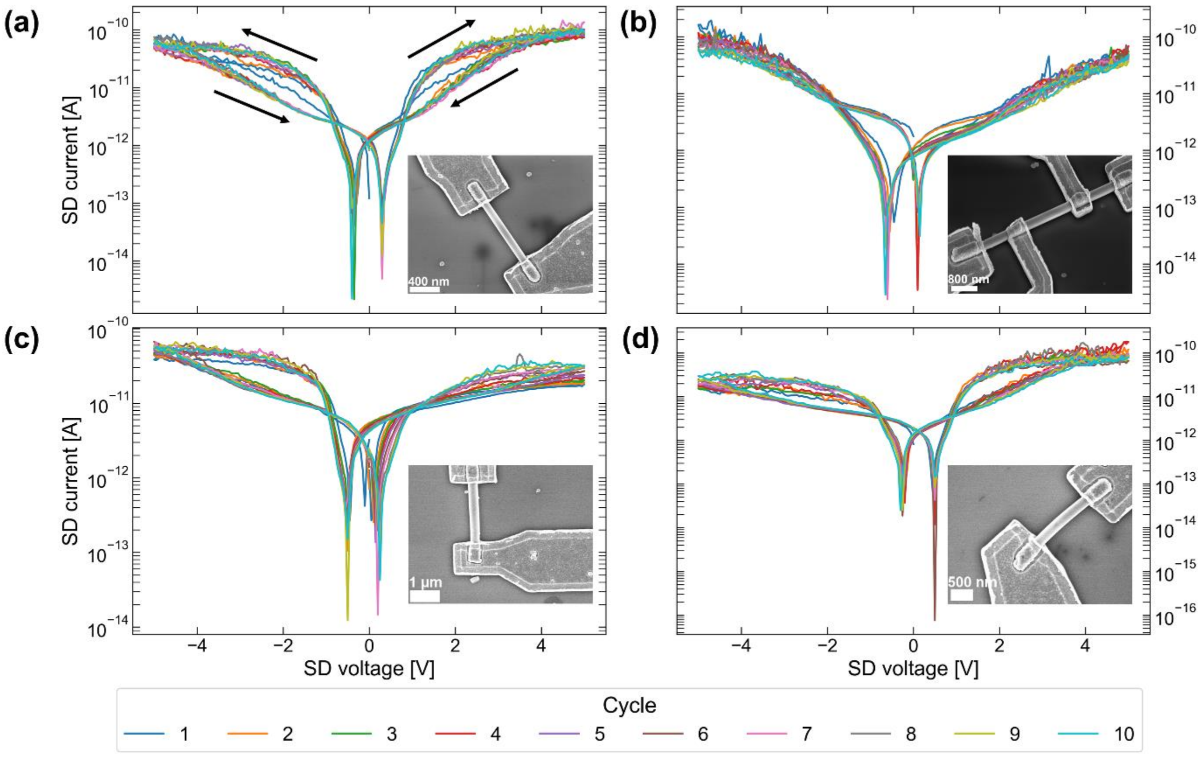

Figure S1. (a), (b), (c), and (d) 10 cycles of photocurrent I-V sweeps for the devices shown in the corresponding insets. Arrows in (a) indicate the sweep direction, which corresponds

identically for all other devices. The device shown in (b) is the same device as in the main manuscript.

REFERENCES

1. Marcal, L. A. B.; Oksenberg, E.; Dzhigaev, D.; Hammarberg, S.; Rothman, A.; Bjorling, A.; Unger, E.; Mikkelsen, A.; Joselevich, E.; Wallentin, J., In Situ Imaging of Ferroelastic Domain Dynamics in CsPbBr3 Perovskite Nanowires by Nanofocused Scanning X-ray Diffraction. ACS Nano 2020, 14 (11), 15973-15982.

2. Storm, K.; Nylund, G.; Borgstrom, M.; Wallentin, J.; Fasth, C.; Thelander, C.; Samuelson, L., Gate-induced fermi level tuning in InP nanowires at efficiency close to the thermal limit. Nano Lett 2011, 11 (3), 1127-30.
